# Reference Intervals and Cut-Off Values for Thyroid Tests in the Croatian Adult Population on the Snibe MAGLUMI X6 Immunoassay Analyzer

**DOI:** 10.3390/diagnostics15182360

**Published:** 2025-09-17

**Authors:** Ivana Lapić, Dragana Šegulja, Željkica Jakoplić, Iva Lukić, Dunja Rogić

**Affiliations:** 1 Department of Laboratory Diagnostics, University Hospital Centre Zagreb, 10 000 Zagreb, Croatia; dsegulja@kbc-zagreb.hr (D.Š.); predstojnik.lab@kbc-zagreb.hr (D.R.); 2 Faculty of Pharmacy and Biochemistry, University of Zagreb, 10 000 Zagreb, Croatia; 3 Medical-Biochemistry Laboratory, Polyclinic Bonifarm, 10 000 Zagreb, Croatia; zjakoplic@bonifarm.hr; 4 Clinical Institute of Laboratory Diagnostics, University Hospital Centre Osijek, 31 000 Osijek, Croatia; iva.lukic@kbco.hr

**Keywords:** thyroid function tests, reference values, immunoassay

## Abstract

**Background/Objectives:** To establish reference intervals (RIs) and cut-off values for thyroid-related tests on the MAGLUMI X6 immunoassay analyzer (Snibe Diagnostic, Shenzhen, China) in an adult Croatian population. **Methods**: This study included 305 healthy individuals who underwent regular preventive medical checkup. The following tests were determined in serum: thyroid-stimulating hormone (TSH), total triiodothyronine (TT3), total thyroxine (TT4), free triiodothyronine (FT3), free thyroxine (FT4), thyroglobulin (Tg), reverse triiodothyronine (revT3), total binding capacity of thyroglobulin (T-uptake), thyroglobulin antibodies (anti-Tg), anti-thyroid peroxidase antibodies (anti-TPO) and thyroid receptor antibodies (TRAb). TSH, TT3, TT4, FT3, FT4, Tg, revT3 and T-uptake results were used for calculating double-sided 95% RIs between the 2.5th and 97.5th percentiles. For anti-Tg, anti-TPO and TRAb, right-sided cut-offs that correspond to the 95th percentile were determined. **Results**: Reference intervals for TSH, TT4, FT3, FT4, Tg, T-uptake and revT3 did not differ by gender (*p* > 0.05) and were 0.77–5.04 mIU/L, 69.9–127.7 nmol/L, 3.84–6.20 pmol/L, 13.8–19.7 pmol/L, 1.8–51.2 µg/L, 0.9–1.2 TBI and 0.44–0.73 ng/mL, respectively. The RI for TT3 was different for males (1.49–2.53 nmol/L) and females (1.43–2.81 nmol/L), *p* = 0.021. A single cut-off for anti-TPO was established (<18 kIU/L). Differences in cut-offs for males and females were obtained for anti-Tg (<72 and <104 kIU/L, respectively) and TRAb (0.6 and 0.9 IU/L, respectively). **Conclusions**: This is the first study to determine RIs for thyroid function tests in Croatian adults on the Snibe analytical platform. The obtained results point out to the use of population- and immunoassay-specific RIs. For TT3, anti-Tg and TRAb gender-specific RIs should be considered.

## 1. Introduction

The post-analytical phase is the final step of the laboratory testing process and consists of results evaluation and reporting [1]. Reference intervals and cut-off values represent crucial information reported alongside every numerical result on the laboratory report. They are regarded as the cornerstone of adequate interpretation and proper clinical use of laboratory test results [2]. A reference interval (RI) is defined as a range of values between the upper and lower reference limit, which comprises 95% of results measured in a statistically adequate sample of the healthy reference population. Instead, cut-off values or decision limits are one-sided thresholds obtained from the healthy reference population data which are used to classify patient results into two categories, based on whether they fall below or above that value [3].

According to the International Organization for Standardization standard 15189:2022 it is obligatory that each laboratory uses RIs and clinical decision limits tailored to the population served [4]. Moreover, RIs and cut-off values are commonly dependent on the analytical method. This is especially pronounced in the field of immunoassays due to the differences in specificity and reactivity of monoclonal antibodies used in the reaction, method of detection and calibration standards used. Therefore, the Clinical and Laboratory Standards Institute (CLSI) in their EP28-A3c guidelines recommends that each laboratory should at least verify the applicability of the reference intervals defined by the manufacturer to its own patient population prior to their use in routine practice or, ideally, establish its own reference intervals [5]. The recommended, most commonly applied approach for establishing RIs is the direct method that implies inclusion of at least 120 healthy individuals for each gender and age group [2,5]. When conducting a RI study, attention should be paid to all pre-analytical and analytical variables that can affect the final result, including biological variability of the analyte, sampling techniques, sample preparation, analytical variation of the method and any other analyte-specific factors [2].

Thyroid disorders encompass a range of conditions affecting the thyroid gland, including functional, autoimmune and malignant disorders. Along with imaging techniques, laboratory measurement of thyroid hormones and thyroid-related antibodies is the cornerstone for proper diagnosis establishment, adequate treatment choice and therapeutic monitoring of patients with thyroid disorders. Therefore, it is essential to use appropriate reference intervals and decision limits.

MAGLUMI immunoassay analyzers developed by Snibe Diagnostic (Shenzhen, China) are being increasingly introduced in European laboratories. However, reference intervals and cut-off values for dedicated assays were established in the Chinese population and can differ due to race and ethnicity. Therefore, the aim of the present study was to establish RIs or cut-off values in the adult Croatian population for a comprehensive panel of thyroid related laboratory tests performed on the MAGLUMI X6 analyzer (Snibe Diagnostic, Shenzhen, China), as follows: thyroid-stimulating hormone (TSH), total triiodothyronine (TT3), total thyroxine (TT4), free triiodothyronine (FT3), free thyroxine (FT4), thyroglobulin, reverse triiodothyronine (revT3), total binding capacity of thyroglobulin (T-uptake), thyroglobulin antibodies (anti-Tg), anti-thyroid peroxidase antibodies (anti-TPO) and thyroid receptor antibodies (TRAb). The calculated RIs and cut-offs were compared to the manufacturer’s recommendations, and partitioning according to gender was done.

## 2. Materials and Methods

### 2.1. Study Setting and Participants

The present study was conducted at the Department of Laboratory Diagnostics, University Hospital Centre Zagreb, Croatia during a one-month period, from February to March 2025. The study population was prospectively recruited from apparently healthy Caucasian individuals who underwent a regular mandatory preventive medical checkup that inevitably includes blood sampling for laboratory testing. The inclusion criteria were formulated as follows: only adults in whom personal medical history and clinical examination were not suggestive of thyroid disorders were included in the study. No study participant was taking any thyroid medications and pregnant women were also excluded. Patients with unmeasurably low TSH (<0.009 mIU/L) or Tg (<0.02 µg/L) were excluded from the study. In addition, for all patients in whom either anti-Tg, anti-TPO, or TRAb tended to be high, samples were re-analyzed on an alternate immunoassay platform used in routine laboratory practice. If the result was above the cut-off used for that immunoassay, those patients were also excluded from the study.

The study was fully conducted following the protocol for direct determination of reference intervals, as defined in the CLSI EP28-A3c document [5].

This study was conducted in accordance with the Declaration of Helsinki and approved by the University Hospital Centre Zagreb Ethics Committee (protocol code: 8.1-25/70-2, 02/013 AG; date of approval: 3 March 2025). Informed consent was obtained from all subjects involved in this study.

### 2.2. Blood Sampling and Laboratory Analyses

Blood sampling was performed between 7 and 9 A.M., and all study participants were in a fasting state and underwent blood sampling after at least 15 min resting time in a sitting position. Blood samples were drawn using a 20-gauge 1.5 in (3.8 cm) needle into a 3 mL serum separator tube with clot activator (Greiner Bio-One, Kremsmünster, Austria). After the samples were left clotting for at least 30 min, serum was obtained by centrifugation for 10 min at 2100× *g*. Samples were stored at 4 °C and all analyses were performed within 8 h from blood sampling.

All analyses were performed on MAGLUMI X6 analyzer (Snibe Diagnostic, Shenzhen, China) which utilizes the chemiluminescence immunoassay technology. For all analyses, dedicated reagents and original manufacturer’s applications were used, and the manufacturer’s instructions were strictly followed. A single reagent lot was used for each evaluated assay and re-calibration was not necessary for either of the assays, as confirmed by acceptable internal quality control results. The validity of the system was monitored daily prior to sample analysis with dedicated commercial quality control samples. Serum samples were analyzed in batches daily after collection.

For evaluation of the initial positivity of anti-Tg, anti-TPO and TRAb, samples were re-analyzed utilizing the Alinity i immunoassay analyzer (Abbott Laboratories, Chicago, IL, USA) with dedicated reagents, applications, calibrators and quality control samples from the same manufacturer.

### 2.3. Statistical Analysis

Data distribution normality was assessed using the Shapiro–Wilk test. The non-parametric percentile method was applied for calculation of RIs and cut-off values, and the Tukey’s method was used for outliers’ testing. For TSH, TT3, TT4, FT3, FT4, Tg, revT3 and T-uptake, double-sided 95% RIs that include the range between the 2.5th and 97.5th percentiles were determined with 90% confidence intervals (CIs). For anti-Tg, anti-TPO and TRAb, right-sided cut-off values that correspond to the 95th percentile were determined, equally with 90% CIs. Assessment of the differences of RIs and cut-offs obtained for males and females, according to age, as well as compared to the manufacturer’s defines ones, was done with the *t*-test, and *p* < 0.05 was considered statistically significant. Statistical analysis was performed in the MedCalc statistical software, version 23.3.2 (MedCalc, Ostend, Belgium).

## 3. Results

**Figure 1:**
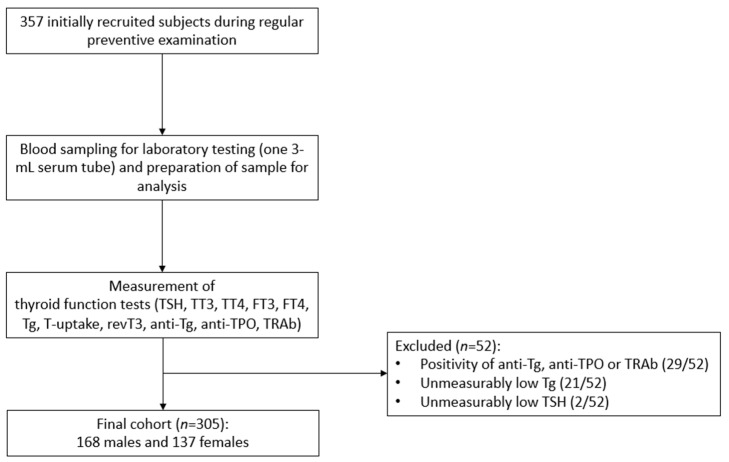
Flow diagram of study participants’ enrollment.

Out of the 357 subjects initially recruited, 52 were excluded according to established criteria and the final cohort consisted of 305 reference individuals (median age 38 years, from 18 to 68 years), of whom 168 (55%) were males. A flow diagram of study participants’ enrollment is presented in Figure 1.

**Figure 2:**
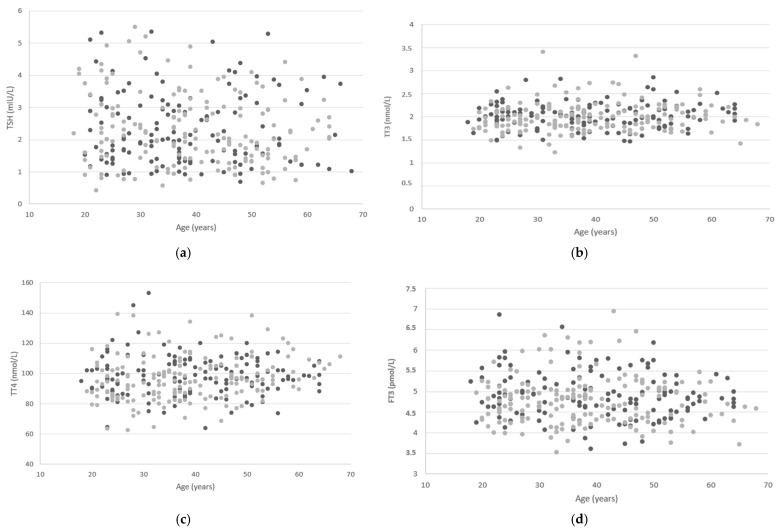

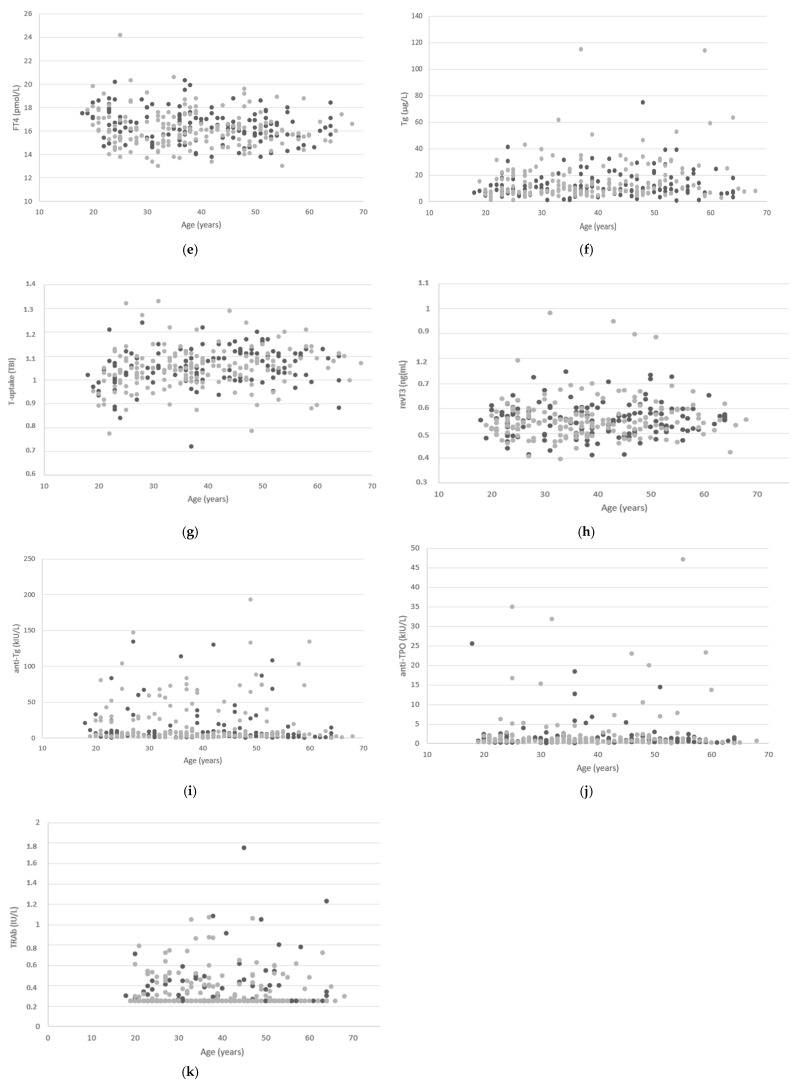
Scatter plots of thyroid function tests’ results per gender in relation to age: (**a**) TSH—thyroid stimulating hormone, (**b**) TT3—total triiodothyronine, (**c**) TT4—total thyroxine, (**d**) FT3—free triiodothyronine, (**e**) FT4—free thyroxine, (**f**) Tg—thyroglobulin, (**g**) T-uptake—total binding capacity of thyroglobulin, (**h**) revT3—reverse triiodothyronine, (**i**) anti-Tg—thyroglobulin antibodies, (**j**) anti-TPO—anti-thyroid peroxidase antibodies, (**k**) TRAb—thyroid receptor antibodies. Dark grey dots correspond to females, while light grey dots correspond to males.

Figure 2 shows scatter plots of thyroid function tests’ results per gender in relation to age.

Reference intervals for TSH, TT3, TT4, FT3, FT4, Tg, revT3 and T-uptake determined in all study participants and separately for males and females, as well as RIs recommended by the manufacturer, are presented in Table 1. No statistically significant differences were found between RIs for males and females, with the exception of TT3 (*p* = 0.021). Statistically significant differences, with *p* < 0.001, between locally established RIs and those recommended by the manufacturer were obtained for TSH, TT3, TT4, FT3 and revT3.

Table 2 reports cut-off values for anti-Tg, anti-TPO and TRAb determined in all study participants and separately for males and females, and the manufacturer’s defined cut-offs. Statistically significant differences between cut-offs for males and females, as well as locally established cut-offs and those recommended by the manufacturer, were obtained for anti-Tg (*p* = 0.005 and *p* < 0.001, respectively) and TRAb (*p* < 0.001 in both cases).

Age-adjusted RIs, i.e., from 18 to 40 years and over 40 years, were determined for the first-line thyroid tests TSH, FT3 and FT4, as reported in Table 3. Statistically significant differences were obtained between RIs for TSH and FT4 (*p* < 0.001 in both cases).

## 4. Discussion

In the present study, RIs and cut-off values for thyroid function tests on the MAGLUMI X6 immunoassay analyzer (Snibe Diagnostic, Shenzhen, China) using the CLIA method by Snibe were established for the first time in the Croatian adult population using the direct method. The obtained results confirmed the importance of establishing local population- and immunoassay-specific RIs and cut-offs. Single RIs and cut-offs can be used for the majority of included tests, with the exception of TT3, anti-Tg and TRAb, for which the use of gender-specific RIs and cut-offs was determined to be more appropriate.

TSH serum level is the single initial most useful test in the evaluation of patients with suspected thyroid function disorders and is also the main test used for monitoring both post-surgical patients as well as those receiving thyroid medications [6,7]. Although not in line with current clinical guidelines, TSH is also being commonly ordered as part of routine medical checkups in asymptomatic subjects [7,8]. In addition, TSH is the only altered thyroid-related parameter in patients with subclinical hypothyroidism, a disorder with reportedly high incidence, between 3.9% and 8.8% [6]. The characteristic mild elevations of TSH in this condition can therefore be missed if inadequate RIs are used. Widespread utilization and demand for TSH tests in our country consequently resulted with its introduction not only into hospital laboratories, but also smaller laboratories serving primary health care centers. Taking into account the well-known differences in immunoassay formulations which can yield differences in results, as well as the effect of race and ethnicity on thyroid function parameters, it is of utmost importance to report TSH results using adequate, population-based and method-specific RIs. Local establishment of RIs has been performed in many settings worldwide and in different age-specific subject groups, revealing variable upper and lower limits. In published studies, lower RI limits range from 0.39 to 0.75 mIU/L, while upper RI limits are found to be between 2.84 and 5.32 mIU/L [9,10,11,12,13,14,15,16]. The RI for TSH calculated in the present study (0.77–5.04 mIU/L) aligns best with the RI obtained in the study by Mirjanic-Azaric et al. [9], who report a RI of 0.75–5.32 mIU/L, despite being measured on a different analytical system, i.e., by Roche Diagnostics. Such matching can be explained by the similar patient population due to geographical proximity. Contrary to that, all RIs deriving from studies conducted in Asian countries are characterized by much lower limits, presenting like a left shift in reference values compared to our results [10,12,13,16], a finding that points out that ethnicity and race do have an effect on TSH levels. This is also supported by the fact that the TSH RI recommended by the manufacturer Snibe, established on the Chinese population, is lower than the one obtained in our study. Additionally, in some studies, indirect rather than direct methods for RIs’ establishment were used with a larger data set and different exclusion criteria, which can be a further source of heterogeneity of the obtained RIs. Importantly, none of the studies published so far were performed on the Snibe platform.

Similarly, when comparing the RIs obtained for thyroid hormones, i.e., FT3, FT4, TT3 and TT4, with other studies, some minor differences can be observed, which can be equally attributed to different patient populations, analytical platforms and approach for RI determination. In our study, among these thyroid hormones, only for FT4 was no statistically significant difference observed between locally obtained RIs and those defined by the manufacturer, Snibe. This finding once again proves the validity of establishing population and method-specific RIs. In addition, the obtained results revealed a statistically significant difference in the RIs between men and women for TT3. The study by Li et al. [16] also evidenced differences in RIs for thyroid hormones between genders, pointing out that gender should be taken into consideration in the diagnostic evaluation of thyroid disorders. However, when evaluating the raw data obtained in the present study, the distribution of TT3 values is rather consistent among the two genders and the differences obtained can be explained by the existence of a minor number of samples with borderline values. Therefore, despite statistical significance, these differences should not be considered clinically significant. Indeed, the universal RI for TT3 balances these slight differences and it can be safely used for interpretation of results since it will not affect clinical decision-making differently.

In the case of thyroglobulin, the obtained RIs are fully concordant with those defined by the manufacturer, but are approximately twice higher than RIs reported across different studies, even the one that was performed in the same geographical region [9,17,18]. Assuming adequate iodine intake, which is the main physiological factor affecting thyroglobulin levels [19], the most logical explanation for the increased upper RI limit compared to other studies but not manufacturer’s recommendations are methodological differences between the used immunoassays. Despite the introduction of a Certified Reference Material (CRM 457), inter-method variability is still relatively high, reportedly being about 30%. This results from a combined effect of the following factors: the use of immunoassay-specific calibration standards, variable epitope specificities of the used antibodies, different efficiency in removing binding proteins and diverse detection principles [20]. Among all the assays assessed within this study, methodological differences were most prominent in the case of thyroglobulin. This finding proves once again that harmonization in the immunoassays field has not been achieved yet and emphasizes the need for assay-specific RIs. Therefore, interchangeable use of assays from different manufacturers is still not recommended and laboratories are advised to clearly state the method used on the laboratory report [21].

The present study defined cut-off values for all three clinically relevant thyroid-associated autoantibodies measured on the Snibe platform. Hereby we proved that, despite the well-known approximately twice higher prevalence of anti-TPO in women than in men [22], a universal cut-off could be used. The study by D’Aurizio et al. [23] dealt with cut-offs for anti-TPO on five different analytical platforms and confirmed the dependance of the cut-offs on the method used, speculating that these differences are a consequence of different coating preparations of the TPO antigen. One of the included analyzers was the MAGLUMI 800 from the manufacturer Snibe, and they demonstrated higher cut-offs for anti-TPO than in our study and equally did not evidence differences between genders. The observed differences can be attributed to different reference populations, since D’Aurizio et al. [23] included only young adults up to 30 years of age. On the contrary, in order to more accurately reflect real patient demographics, our study comprised subjects aged up to 65. Results for other two relevant autoantibodies measured within our study clearly indicate that for reporting of anti-Tg and TRAb, gender-adjusted cut-offs should be considered, in both cases being higher in females. This finding was expected due to the well-known higher prevalence of autoimmune thyroid disorders in women [22]. While the universal cut-off for anti-Tg proposed by Mirjanic-Azaric et al. [9] is in line with the one obtained in our study, cut-offs obtained for TRAb were significantly lower than the one recommended by the manufacturer and those reported in the study by Tanticharoenkarn et al. [12] performed on the Roche platform. Finally, to the best of our knowledge, this is the first study to report locally established RIs for revT3 and T-uptake, tests which are not yet included in any clinical guidelines for thyroid management but can serve as valuable diagnostic adjuncts for elucidation of specific cases.

This study has some limitations. Firstly, this is a single-center study performed during a relatively short time period of one month, including a consecutive sample of apparently healthy subjects who underwent mandatory medical check-up. Secondly, study participants did not undergo thyroid ultrasound to exclude any possible underlying thyroid disorders. However, none of the included study participants were aware of any thyroid disorder and strict exclusion criteria were applied to exclude outliers that were identified by laboratory analysis. In addition, before exclusion, those results were confirmed by the routinely used method. Therefore, we believe that the calculated RIs and cut-offs herein accurately reflect the expected values in the healthy Croatian adult population.

In conclusion, this is the first study to report RIs and cut-offs for all thyroid function tests available in the Snibe portfolio, not only in Croatia but the whole continent of Europe. The observed differences in calculated RIs compared to the ones recommended by the manufacturer once again point out to the influence of race and ethnicity on results of thyroid function tests and confirm the validity of the local establishment of RIs. Further refinement of laboratory results reporting can be achieved by the use of gender-specific RIs and cut-offs, especially in cases of thyroid-associated autoantibodies. The RIs and cut-offs established herein can serve as a valuable starting point in laboratories who are considering introduction of Snibe analyzers into routine practice, especially those in nearby geographical areas and with a predominantly Caucasian patient population. If verified on the local population through a brief protocol as defined in the CLSI EP28-A3 document [5], those assays can be safely introduced into routine practice. Ultimately, locally established RIs and cut-offs are a valuable post-analytical tool that streamlines the adequate interpretation of laboratory results and hence contributes to favorable outcomes and patient safety.

## Figures and Tables

**Table 1:** Universal and gender-specific reference intervals established in this study compared to reference intervals defined by the manufacturer, Snibe Diagnostic.

	Locally Established RIs				RIs Recommended by the Manufacturer Snibe Diagnostic	
	All Study Participants (*n* = 305)	Males (*n* = 168)	Females (*n* = 137)	*p* Value *	Both Genders	*p* Value **
TSH (mIU/L)	0.77–5.04	0.77–5.18	0.76–4.97	0.207	0.30–4.50	<0.001
TT3 (nmol/L)	1.49–2.71	1.49–2.53	1.43–2.81	0.021	0.75–2.10	<0.001
TT4 (nmol/L)	69.9–127.7	64.4–126.6	72.8–136.2	0.096	64.1–166.7	<0.001
FT3 (pmol/L)	3.84–6.20	3.89–5.96	3.74–6.51	0.652	3.07–6.45	<0.001
FT4 (pmol/L)	13.8–19.7	13.8–19.8	13.8–19.8	0.202	11.5–22.4	0.239
Thyroglobulin (μg/L)	1.8–51.2	1.3–48.6	2.5–56.3	0.850	1.4–50.7	0.647
T-uptake (TBI)	0.9–1.2	0.9–1.2	0.9–1.3	0.067	0.8–1.3	1.000
revT3 (ng/mL)	0.44–0.73	0.44–0.71	0.41–0.85	0.276	0.31–0.95	<0.001

* *p* value obtained by the *t*-test used for assessment of statistically significant differences between reference intervals for males and females. ** *p* value obtained by the *t*-test used for assessment of statistically significant difference between locally established universal reference intervals and those recommended by the manufacturer, Snibe Diagnostic.

**Table 2:** Universal and gender-specific cut-off values established in this study compared to cut-off values defined by the manufacturer, Snibe Diagnostic.

	Locally Established Cut-Offs				Cut-offs Recommended by the Manufacturer, Snibe Diagnostic	
	All Study Participants (*n* = 305)	Males (*n* = 168)	Females (*n* = 137)	*p* Value *	Both Genders	*p* Value **
anti-Tg (kIU/L)	<79	<72	<104	0.005	<95	<0.001
anti-TPO (kIU/L)	<18	<5	<24	0.330	<10	0.213
TRAb (IU/L)	<0.7	<0.6	<0.9	<0.001	<1.5	<0.001

*p value obtained by the *t*-test used for assessment of statistically significant differences between cut-off values for males and females. ** *p* value obtained by the *t*-test used for assessment of statistically significant difference between locally established universal cut-off values and those recommended by the manufacturer, Snibe Diagnostic.

**Table 3:** Age-adjusted reference intervals for TSH, FT3 and FT4.

	18–40 Years (*n* = 177)	>40 Years (*n* = 128)	*p* Value *
TSH (mIU/L)	0.77–5.12	0.77–4.37	<0.001
FT3 (pmol/L)	3.88–6.20	3.77–6.19	0.384
FT4 (pmol/L)	13.8–20.2	13.8–18.8	<0.001

* *p* value obtained by the *t*-test used for assessment of statistically significant differences between reference intervals for the two age groups.

## Data Availability

The raw data supporting the conclusions of this article will be made available by the authors on request.

## Abbreviations

The following abbreviations are used in this manuscript:

RI	reference interval
CLSI	Clinical and Laboratory Standards Institute
TSH	thyroid-stimulating hormone
TT3	total triiodothyronine
TT4	total thyroxine
FT3	free triiodothyronine
FT4	free thyroxine
Anti-Tg	thyroglobulin antibodies
Anti-TPO	anti-thyroid peroxidase antibodies
TRAb	thyroid receptor antibodies
revT3	reverse triiodothyronine
T-uptake	total binding capacity of thyroglobulin
IU	international unit
CI	confidence interval
TBI	thyroxine-binding index
